# Supplementary material for: Regulation of priming effect by soil organic matter stability over a broad geographic scale
Source: Nat Commun. 2019 Nov 8;10:5112. doi: 10.1038/s41467-019-13119-z (PMC6841703; doi:10.1038/s41467-019-13119-z)
Supplement: Supplementary file 1 — Supplementary Information [file 41467_2019_13119_MOESM1_ESM.pdf]

Supplementary Information for

Regulation of priming effect by soil organic matter stability over a broad geographic scale

Chen et al.

Supplementary Methods

Carbonate addition experiments

To explore whether and how much carbonates can be released as carbon dioxide (CO_2), we conducted two carbonate addition experiments to quantify the contribution of soil carbonates to the CO_2 flux. For the first experiment, quartz sands (20 g) were incubated in triplicate with two $CaCO_3$ addition treatments (control and $CaCO_3$). As a supplement, the second experiment was conducted for one typical soil without carbonates and included two $CaCO_3$ treatments (control, ^{13}C-labelled $CaCO_3$ (98 at% ^{13}C)). Given that the average inorganic C content was around 8 mg g^{-1} soil for our soil samples (Supplementary Data 1), the rate of inorganic C addition was set to 8 mg g^{-1} soil (~66.7 mg $CaCO_3$ g^{-1} soil) in both experiments. $CaCO_3$ was evenly mixed with either quartz sands or soils before 7-day's pre-incubation. The CO_2 production was then measured during the subsequent 30-day incubation at a fixed soil moisture level (60% of the water holding capacity).

The fraction of CO_2-C derived from the added ^{13}C-$CaCO_3$ (f_{CaCO3}) in the second experiment was determined by Supplementary Equation (1).

$$f_{CaCO_3} = (at\%_{treat} - at\%_{SOC})/(at\%_{CaCO_3} - at\%_{SOC}) \quad (1)$$

where $at\%_{treat}$, $at\%_{control}$, $at\%_{CaCO3}$ and $at\%_{SOC}$ are the C isotope compositions (in at% ^{13}C) of CO_2 from the ^{13}C-$CaCO_3$-treated soil, control soil, added ^{13}C-$CaCO_3$ and soil organic carbon (SOC), respectively.

Phospholipid fatty acid (PLFA) and enzyme activity analyses

PLFAs were extracted from the soil following the protocol[1]. FAME 19:0 (Matreya Inc., State College, PA, USA) was used as standard for the quantitation of each sample. Agilent 6890 GC (Agilent Technologies, Santa Clara, CA, USA) and the MIDI Sherlock Microbial Identification System (MIDI Inc., Newark, DE, USA) were applied to perform qualitative and quantitative fatty acid analyses. PLFAs specific to bacteria (i14:0, a15:0, i15:0, i16:0, 16:1w7c, a17:0, cy17:0, i17:0, 18:1 w7c, cy19:0) and fungi (18:2 w6,9c) were quantified[2]. The microbial community structure was assessed using the fungi/bacteria ratio.

The enzyme activities were assayed using fluorometric techniques by constructing calibration curves for each sample[3]. The 4-methylumbelliferone (MUB) was used as standard for β-1,4-glucosidase (BG), β-1,4-N-acetylglucosaminidase (NAG) and phosphatase (AP), and the 7-amino-4-methylcoumarin (AMC) was chosen as standard for leucine aminopeptidase (LAP). For specific enzyme activity test, 200 μl soil slurry was dispensed into 96-well black microplate, with sixteen replicates for each soil sample. Then the soil slurry was mixed with 50 μl fluorometric substrate solution (200 μM, saturating concentration, 4-MUB-β-D-glucoside for BG, 4-MUB-N-acetyl-β-D-glucosaminide for NAG, L-leucine-7-amido-4-methylcoumarin for LAP and 4-MUB-phosphate for AP) and incubated for 6 h (BG, NAG and LAP) or 2 h (AP) at 25 °C. After incubation, the reactions were stopped by adding NaOH. The amount of fluorescence was finally determined using a fluorometer (Beckman Coulter D TX 880,

Indianapolis, IN, USA) with 365-nm excitation and 450-nm emission.

The procedure of chemical extractions and biomarker analysis

Approximately 6-10 g freeze-dried soil was extracted with 20 ml dichloromethane, dichloromethane: methanol (1:1; v/v) and methanol, respectively[4]. The combined extracts were then concentrated by rotary evaporation and finally dried under N_2 gas.

For extraction of hydrolysable lipids, 15 ml methanolic KOH (1 M) and 3-5 g air-dried soil residues (after the solvent extraction) were added into Teflon-lined bombs to ensure a seal, and heated at 100 °C for 3 h. After cooling, the extracts were acidified to pH 1.0 with HCl (6 M). Hydrolysable lipids were then obtained by liquid-liquid extraction with 20 ml ethyl acetate three times, concentrated by rotary evaporation, and dried with N_2 gas. The extracts were methylated with 14% diazomethane (reaction for 90 min at 70 °C), recovered by liquid-liquid extraction with hexane three times, and dried under N_2 gas[4].

For lignin-derived phenol analysis, 3-5 g soil residues (after the solvent extraction) were mixed with 1 g CuO and 100 mg ammonium iron (II) sulfate hexahydrate into Teflon bombs. Then 15 ml NaOH (2 M) was added into the bombs, and heated at 170 °C for 2.5 h. The extracts were acidified to pH 1.0 with HCl (6 M) and kept at room temperature for 1 h in the dark. After centrifuging at 3000 rpm for 15 min, the supernatant was transferred to a funnel and recovered by liquid-liquid extraction with

20 ml ethyl acetate three times. The ethyl acetate extracts were then concentrated by rotary evaporation and dried under N_2 gas[4].

All biomarkers from solvent extracts, base hydrolysis and CuO oxidation were converted to trimethylsilyl (TMS) derivatives by reaction with 100 μl N,O-bis-(trimethylsilyl) trifluoroacetamide (BSTFA) and 50 μl pyridine at 60 ºC for 2 h. After cooling, dichloromethane was added to dilute to 1 ml for solvent extracts, and ethyl acetate was added to dilute to 1 ml for base hydrolysis and lignin-derived phenols[4]. The biomarkers from solvent extracts and base hydrolysis were identified and quantified by a gas chromatography-mass spectrometry (GC-MS) (Agilent 7890A-5973N, Palo Alto, CA, USA). The biomarkers from CuO oxidation were identified by GC-MS and quantified by an Agilent 7890A GC coupled to an FID. Data were acquired and processed with the GC ChemStation software (Rev. B.04.02). The concentration of individual compounds was normalized to the SOC content.

Supplementary Figures

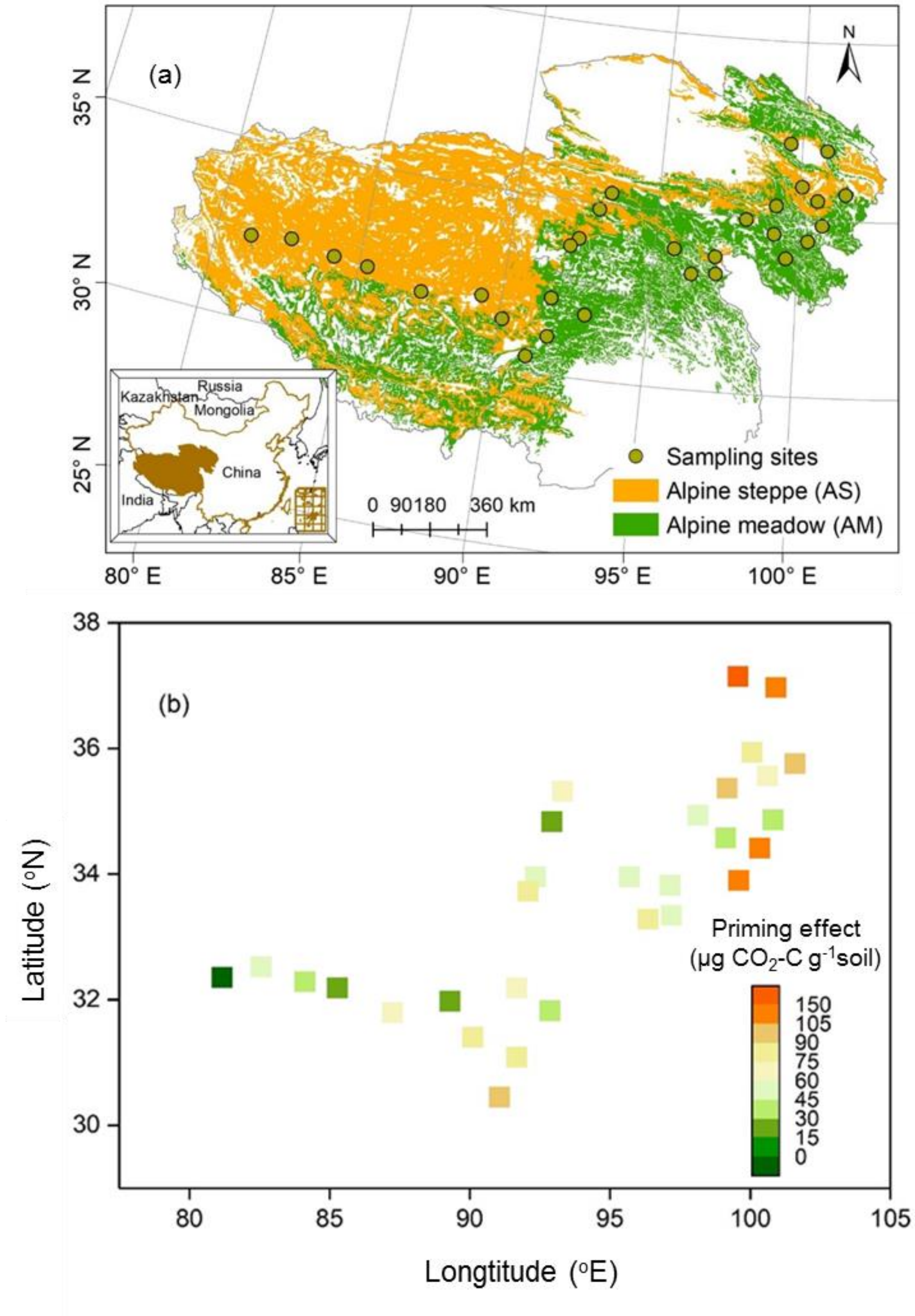

Supplementary Figure 1. Geographic distributions of sampling sites and spatial patterns of the priming effect . For panel (a), the sampling sites are shown on the background of a regional vegetation map, which is derived from China's Vegetation Atlas with a scale of 1: 1 000 000[5]. Panel (b) showed the variations of the priming effect along a 2200 km grassland transect on the Tibetan Plateau.

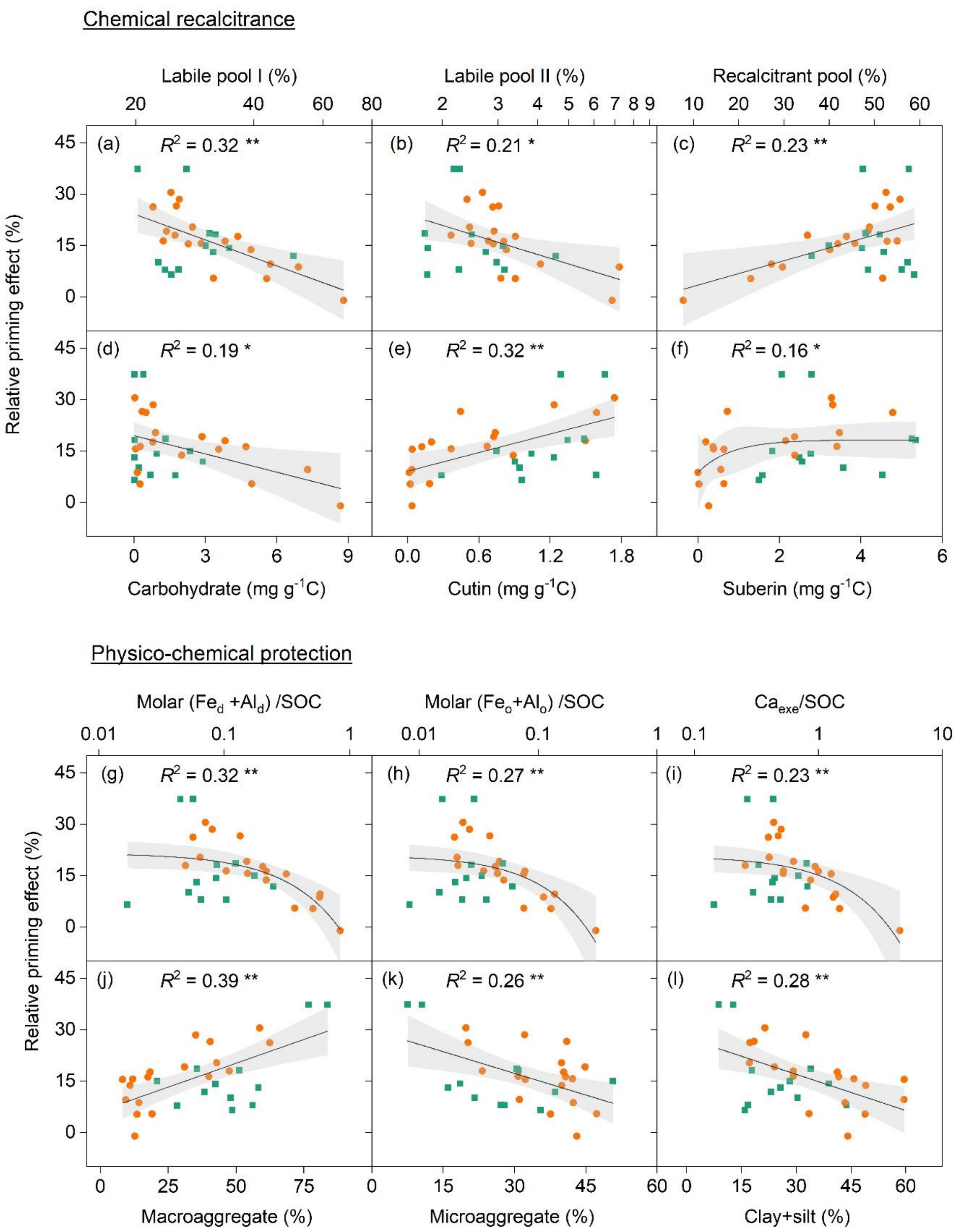

Supplementary Figure 2. Relationships of the relative priming effect (as percentage of basal respiration) with soil organic matter chemical recalcitrance and physico-chemical protection. Soil organic matter chemical recalcitrance is characterized by the proportion of labile pool I (a), labile pool II (b), and recalcitrant pool (c), the relative

abundances of carbohydrate (d), cutin-derived compounds (e) and suberin-derived compounds (f). Physico-chemical protection is reflected by the molar ratio of free Fe/Al oxides (g) and amorphous Fe/Al oxides (h) to SOC, ratio of exchangeable Ca to SOC (i), and SOC proportion in macroaggregates (j), microaggregates (k), and clay+silt fractions (l). A base-10 log scale is used for the x axes of panel (a-b) and (g-i). The orange dots and green squares represent data derived from alpine steppe and alpine meadow, respectively. The solid lines represent the fitted ordinary least squares model, and the grey areas correspond to 95% confidence intervals. * and ** indicate significant correlation between the relative priming effect and the corresponding variables at the 0.05 and 0.01 levels, respectively.

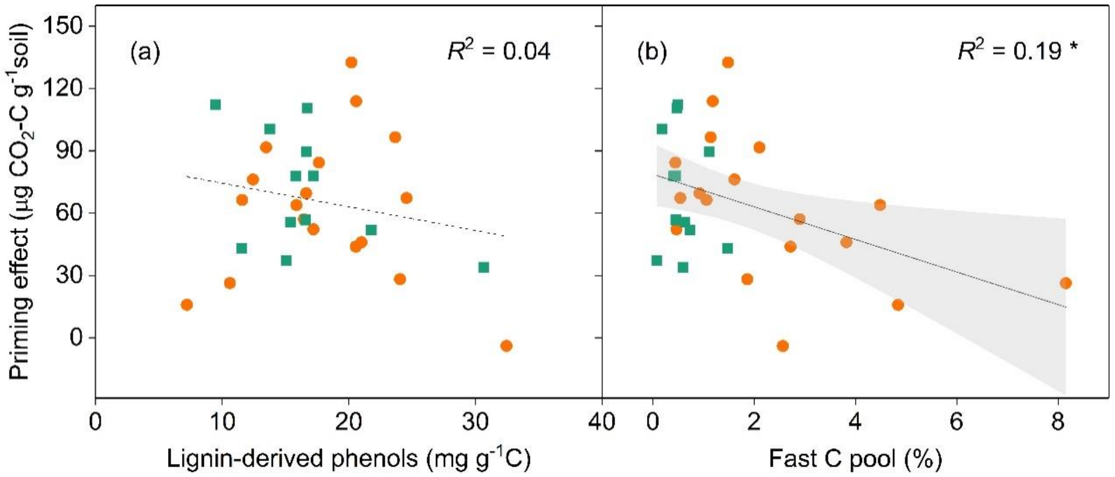

Supplementary Figure 3. Relationships of priming effect with lignin-derived phenols (a) and the proportion of fast carbon pool inversed from the two-pool carbon decomposition model (b). The dashed line indicates an insignificant relationship between the two variables, while the solid line represents the fitted ordinary least squares model, with the grey area corresponding to 95% confidence intervals. * denotes significant correlation between priming effect and the proportion of fast C pool at the 0.05 level. The orange dots and green squares represent data derived from alpine steppe and alpine meadow, respectively.

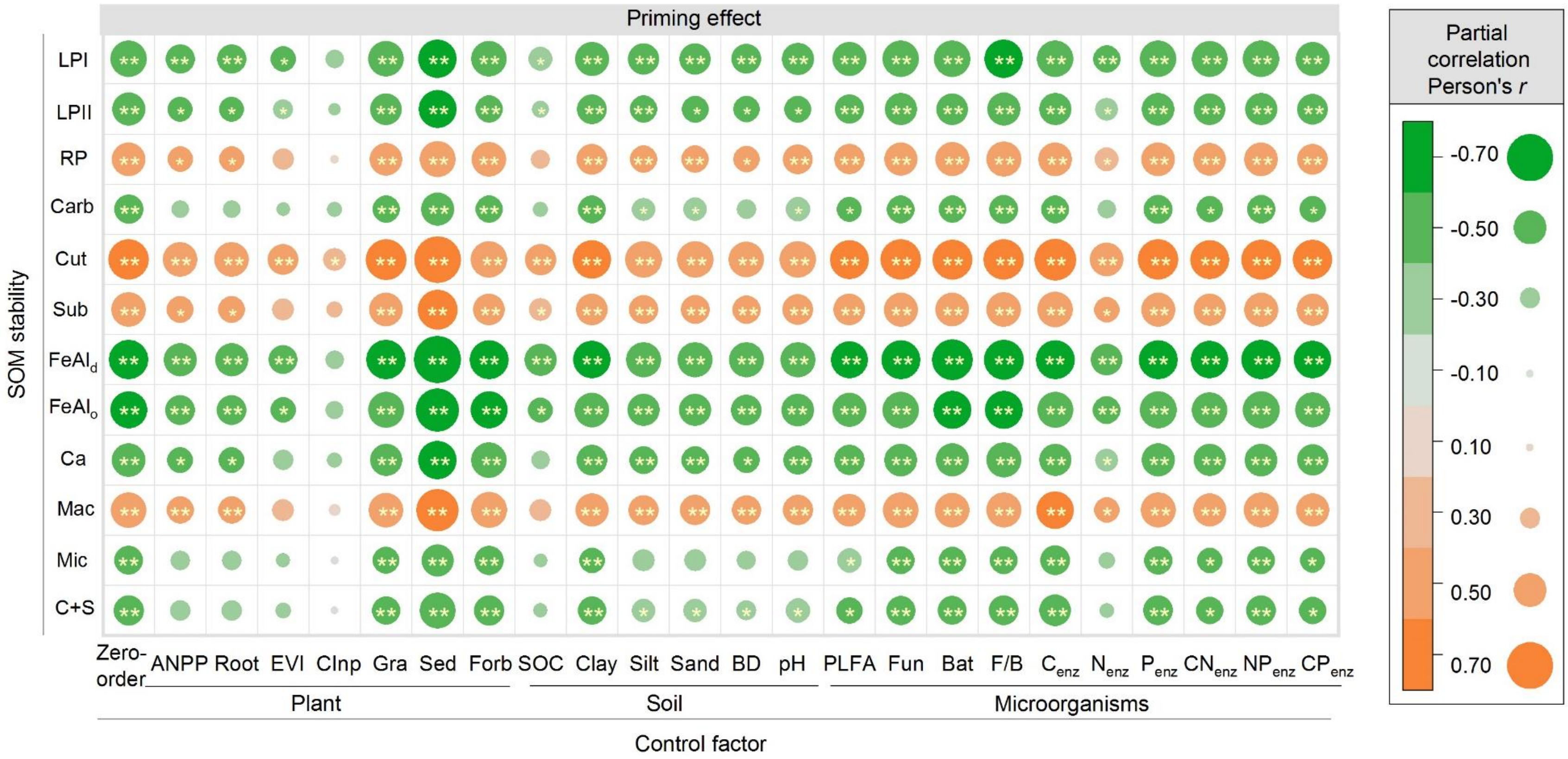

Supplementary Figure 4. Partial correlations between priming effect and soil organic matter stability after controlling plant, soil and microbial properties. The x-axis shows the zero-order (without controlling any factors) and the factors being controlled. The y-axis shows the factors of which the correlations with priming effect are examined. The size and colour of the circle indicate the strength and sign of the correlation. Differences in the circle size and colour between zero-order and controlled factor indicate the level of dependency

of the correlation between priming effect and the examined factor on the controlled variable (No change in circle size and colour between controlled factor and zero-order = no dependency; decrease/increase of circle size and colour intensity = loss /gain of correlation). LPI, labile pool I; LPII, labile pool II; RP, recalcitrant pool; Carb, carbohydrate; Cut, cutin-derived compound; Sub, suberin-derived compound; $FeAl_d$, molar ratio of free Fe/Al oxides to SOC; $FeAl_o$, molar ratio of amorphous Fe/Al oxides to SOC; Ca, ratio of exchangeable Ca to SOC; Mac, proportion of C occluded in macroaggregates; Mic, proportion of C protected by microaggregates; C+S, proportion of C associated with clay+silt fractions. ANPP, aboveground net primary productivity; Root, root biomass; EVI, enhanced vegetation index; CInp, C input amount; Gra, relative coverage of grass; Sed, relative coverage of sedge; Forb, relative coverage of forb; SOC, soil organic carbon content; BD, bulk density; PLFA, total PLFAs; Fun, fungal PLFAs; Bat, bacterial PLFAs; F/B, fungi/bacteria ratio; C_{enz}, C-acquire enzyme activity; N_{enz}, N-acquire enzyme activity; P_{enz}, P-acquire enzyme activity; CN_{eny}, C:N ratio of enzyme activity; NP_{enz}, N:P ratio of enzyme activity; CP_{enz}, C:P ratio of enzyme activity. * $p < 0.05$, ** $p < 0.01$.

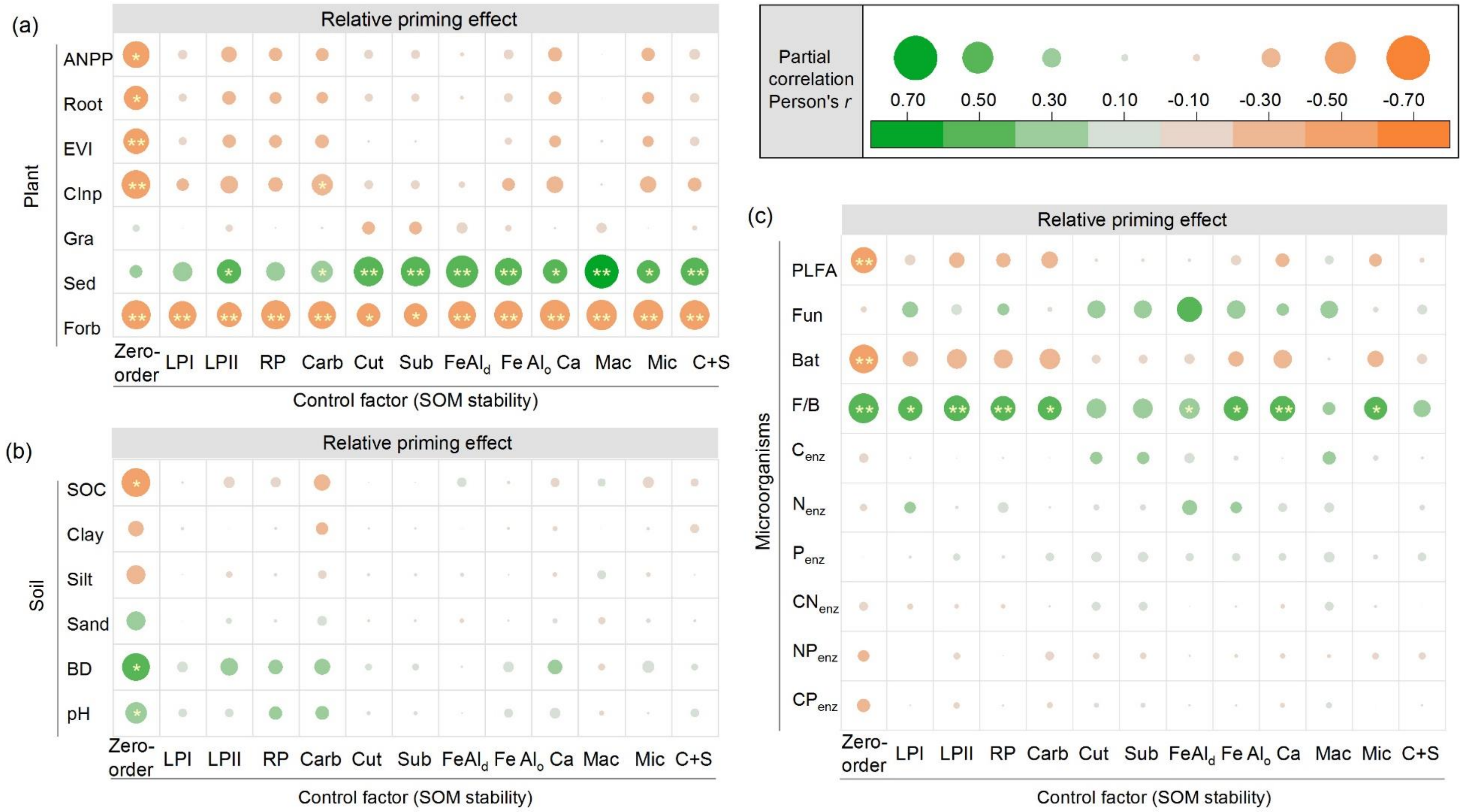

Supplementary Figure 5. Partial correlations between the relative priming effect (as percentage of basal respiration) and the plant, soil and microbial properties after controlling SOM stability. The x-axis shows the zero-order (without controlling any factors) and the

factors being controlled. The y-axis shows the factors (a. plant, b. soil, c. microbial properties) of which the correlations with the priming effect are examined. The size and colour of the circles indicate the strength and sign of the correlation. Differences in circle size and colour between the zero-order and controlled factors indicate the level of dependency of the correlation between the priming effect and the examined factor on the controlled variable (no change in circle size and colour between the controlled factor and zero-order = no dependency; a decrease/increase in circle size and colour intensity = loss /gain of correlation). LPI, labile pool I; LPII, labile pool II; RP, recalcitrant pool; Carb, carbohydrate; Cut, cutin-derived compound; Sub, suberin-derived compound; $FeAl_d$, molar ratio of free Fe/Al oxides to SOC; $FeAl_o$, molar ratio of amorphous Fe/Al oxides to SOC; Ca, ratio of exchangeable Ca to SOC; Mac, proportion of C occluded in macroaggregates; Mic, proportion of C protected by microaggregates; C+S, proportion of C associated with clay+silt fractions. ANPP, aboveground net primary productivity; Root, root biomass; EVI, enhanced vegetation index; CInp, C input amount; Gra, relative coverage of grass; Sed, relative coverage of sedge; Forb, relative coverage of forb; SOC, soil organic carbon content; BD, bulk density; PLFA, total PLFAs; Fun, fungal PLFAs; Bat, bacterial PLFAs; F/B, fungi/bacteria ratio; C_{enz}, C-acquire enzyme activity; N_{enz}, N-acquire enzyme activity; P_{enz}, P-acquire enzyme activity; CN_{eny}, C:N ratio of enzyme activity; NP_{enz}, N:P ratio of enzyme activity; CP_{enz}, C:P ratio of enzyme activity. * $p < 0.05$, ** $p < 0.01$.

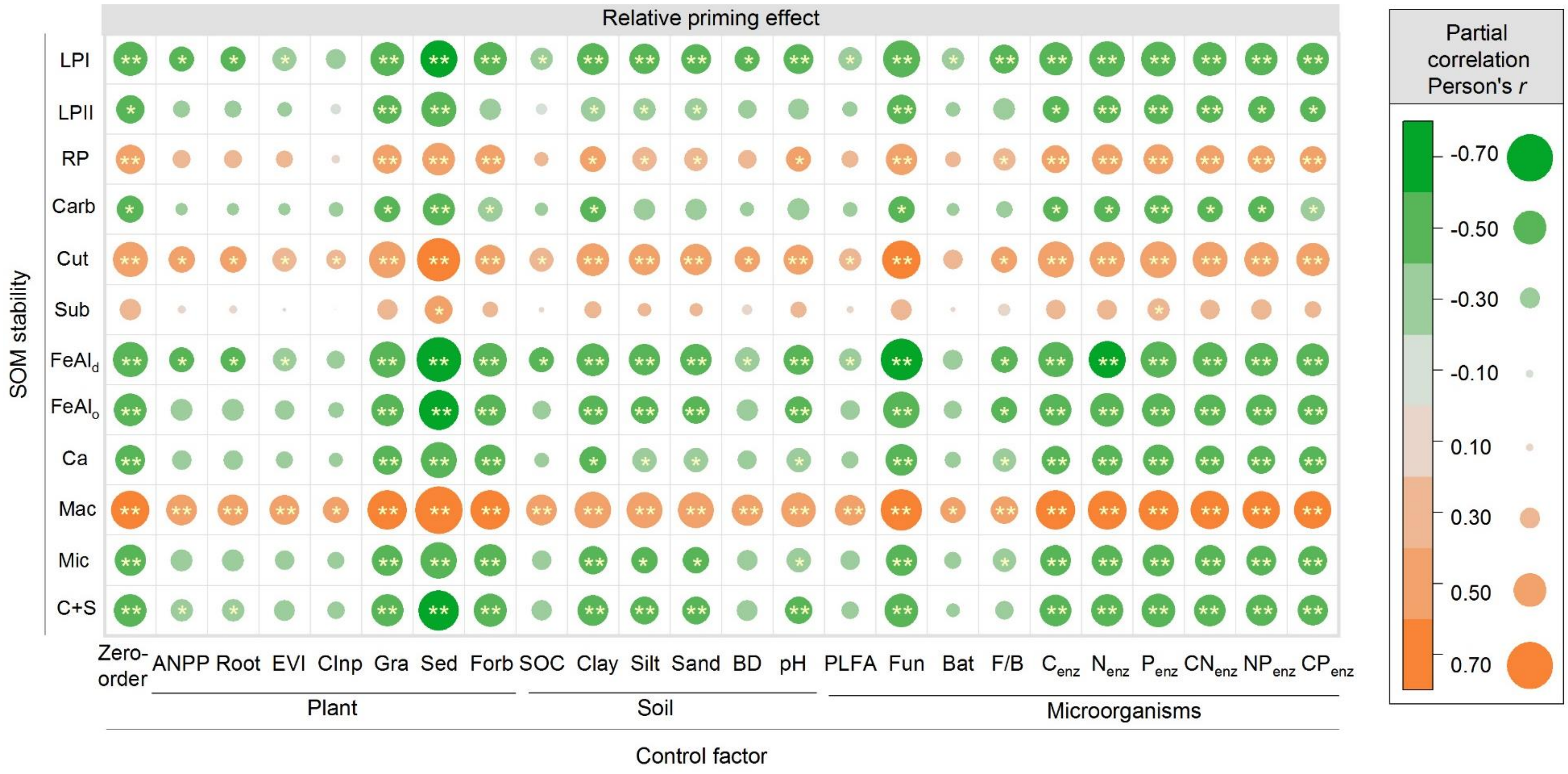

Supplementary Figure 6. Partial correlations between the relative priming effect (as percentage of basal respiration) and soil organic matter stability after controlling plant, soil and microbial properties. LPI, labile pool I; LPII, labile pool II; RP, recalcitrant pool; Carb, carbohydrate; Cut, cutin-derived compound; Sub, suberin-derived compound; FeAl$_d$, molar ratio of free Fe/Al oxides to SOC; FeAl$_o$,

molar ratio of amorphous Fe/Al oxides to SOC; Ca, ratio of exchangeable Ca to SOC; Mac, proportion of C occluded in macroaggregates; Mic, proportion of C protected by microaggregates; C+S, proportion of C associated with clay+silt fractions. ANPP, aboveground net primary productivity; Root, root biomass; EVI, enhanced vegetation index; CInp, C input amount; Gra, relative coverage of grass; Sed, relative coverage of sedge; Forb, relative coverage of forb; SOC, soil organic carbon content; BD, bulk density; PLFA, total PLFAs; Fun, fungal PLFAs; Bat, bacterial PLFAs; F/B, fungi/bacteria ratio; C_{enz}, C-acquire enzyme activity; N_{enz}, N-acquire enzyme activity; P_{enz}, P-acquire enzyme activity; CN_{eny}, C:N ratio of enzyme activity; NP_{enz}, N:P ratio of enzyme activity; CP_{enz}, C:P ratio of enzyme activity. * $p < 0.05$, ** $p < 0.01$.

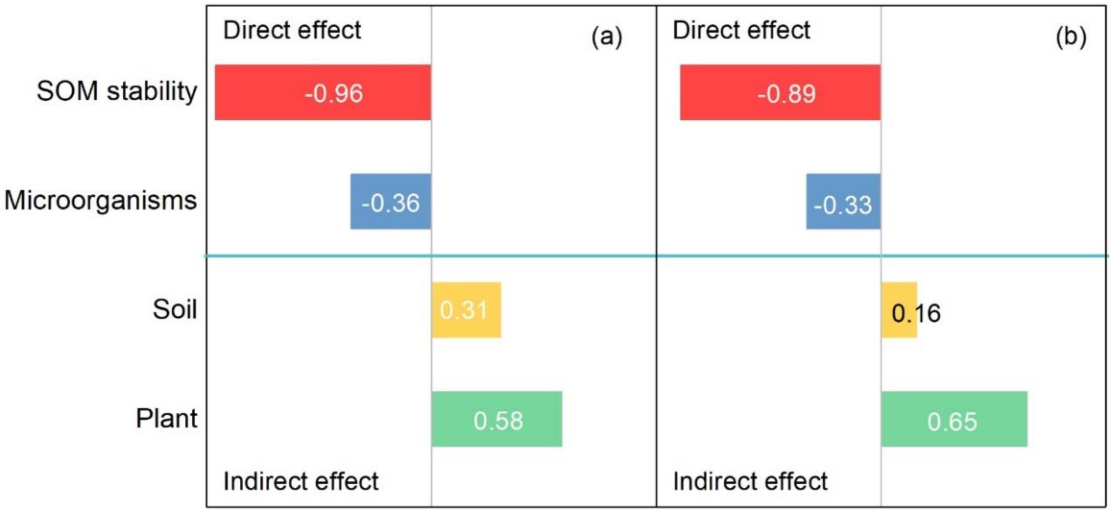

Supplementary Figure 7. Standardized direct and indirect effects of plant, soil and microbial properties, and soil organic matter stability on priming effect. The structural equation modelling was conducted for two forms of priming effect: (**a**) absolute priming intensity; (**b**) relative priming intensity. The numbers adjacent to bar are the standardized coefficients in structural equation modelling.

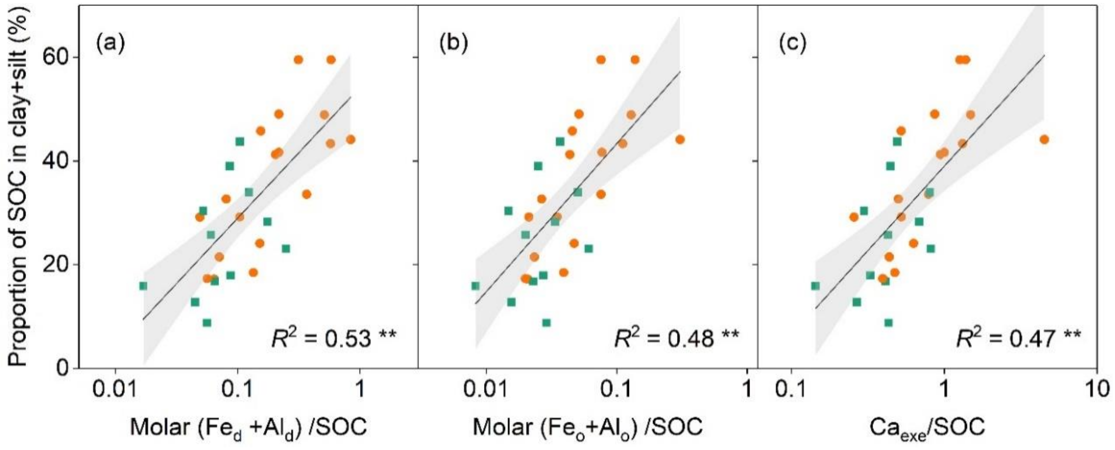

Supplementary Figure 8. Relationships of the proportion of soil organic carbon associated with clay+silt fractions (<53 μm) with the molar ratios of free Fe/Al oxides (a) and amorphous Fe/Al oxides (b) to soil organic carbon (SOC), and ratio of exchangeable calcium (Ca_{exe}) to SOC (c). A base-10 log scale is used for the x axes. The orange dots and green squares represent data derived from alpine steppe and alpine meadow, respectively. ** indicates significant correlation at the 0.01 level.

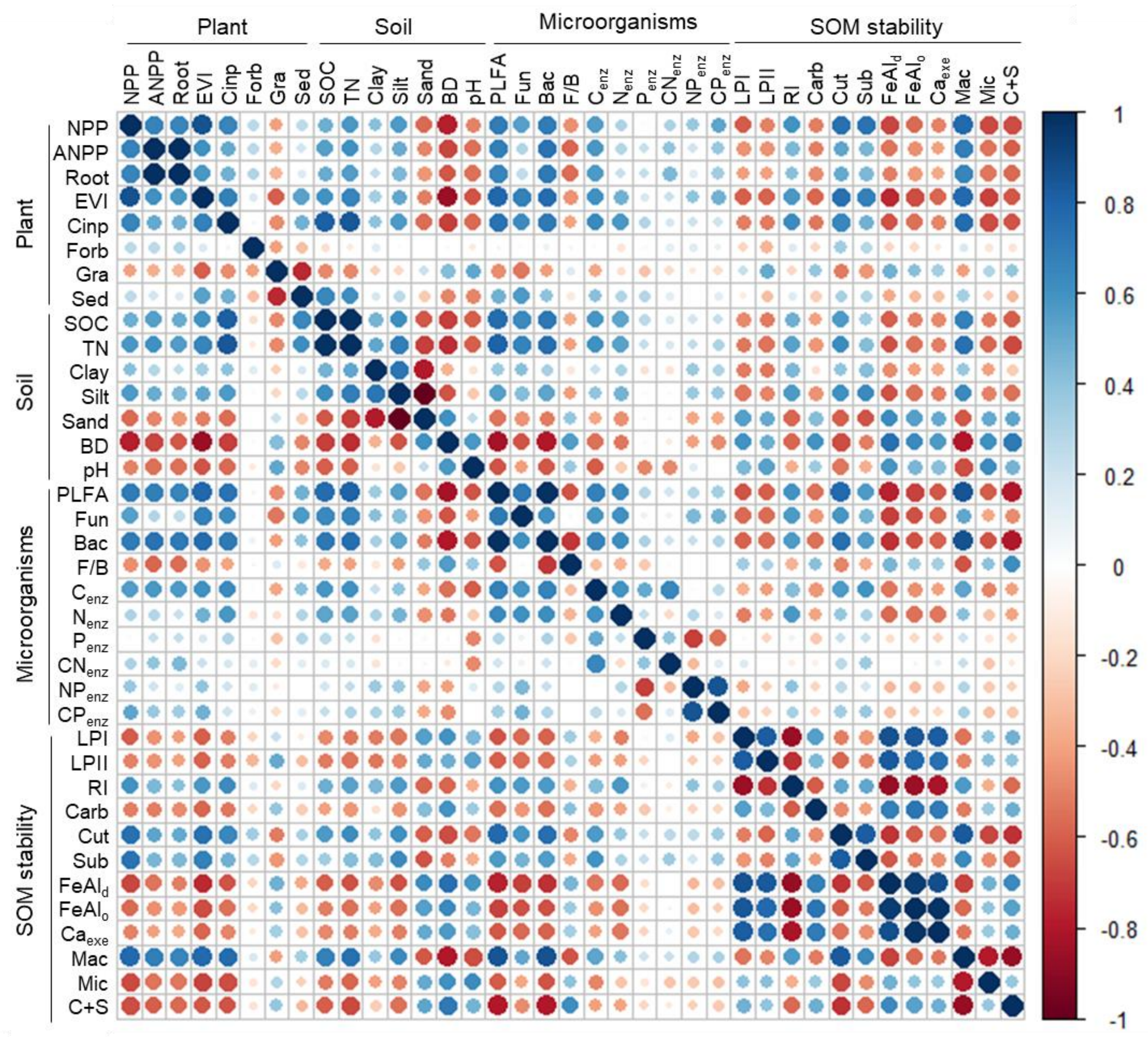

Supplementary Figure 9. Heat map showing correlations among all examined variables. NPP, net primary productivity; ANPP, aboveground net primary productivity; Root, root biomass; EVI, enhanced vegetation index; Cinp, C input amount; Gra, relative coverage of grass; Sed, relative coverage of sedge; Forb, relative coverage of forb; SOC, soil organic carbon content; TN, total nitrogen content; BD, bulk density; PLFA, total PLFAs; Fun, fungal PLFAs; Bat, bacterial PLFAs; F/B, fungi/bacteria ratio; C_{enz}, C-acquire enzyme activity; N_{enz}, N-acquire enzyme activity; P_{enz}, P-acquire enzyme activity; CN_{eny}, C:N ratio

of enzyme activity; NP_{enz}, N:P ratio of enzyme activity; CP_{enz}, C:P ratio of enzyme activity; LPI, labile pool I; LPII, labile pool II; RP, recalcitrant pool; Carb, carbohydrate; Cut, cutin-derived compound; Sub, suberin-derived compound; $FeAl_d$, molar ratio of free Fe/Al oxides to SOC; $FeAl_o$, molar ratio of amorphous Fe/Al oxides to SOC; Ca, ratio of exchangeable Ca to SOC; Mac, proportion of C occluded in macroaggregates; Mic, proportion of C protected by microaggregates; C+S, proportion of C associated with clay+silt fractions.

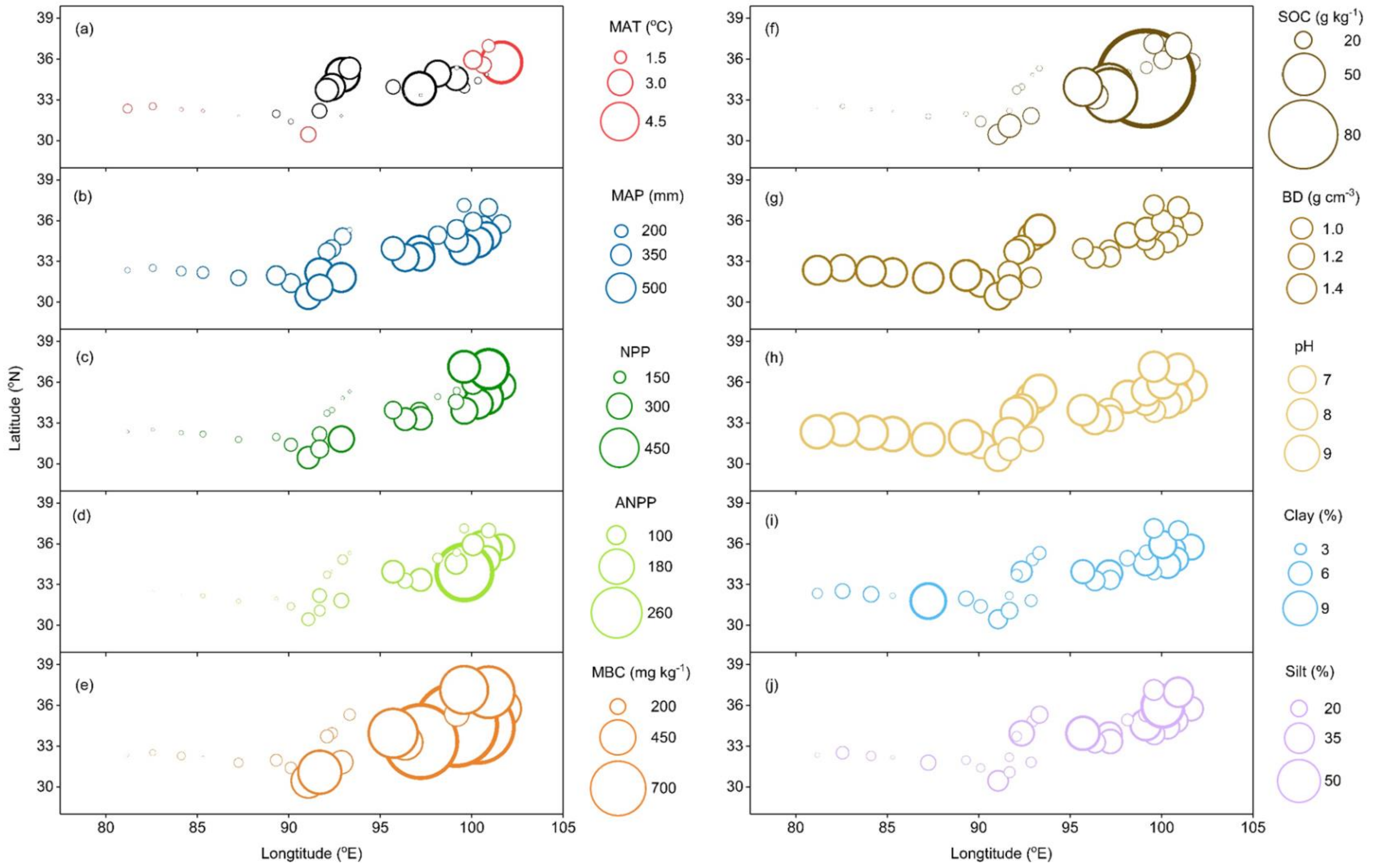

Supplementary Figure 10. Bubble plot representing spatial patterns of climate, plant, soil and microbial properties along the 2200 km grassland transect on the Tibetan Plateau. MAT (a), MAP (b), NPP (c), ANPP (d), MBC (e), SOC (f), BD (g), pH (h), clay (i) and silt (j). MAT, mean annual

temperature; MAP, mean annual precipitation, NPP, net primary productivity; ANPP, aboveground net primary productivity; MBC, microbial biomass carbon; SOC, soil organic carbon content; BD, bulk density. In panel (a), black bubble indicates the negative value, while red bubble indicates the positive value.

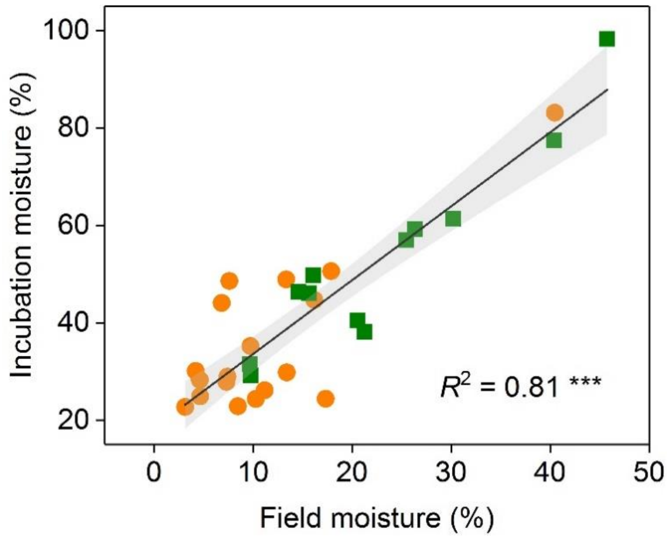

Supplementary Figure 11. Relationship between incubation moisture and field moisture across 30 sampling sites along a 2200 km grassland transect on the Tibetan Plateau. The orange dots and green squares represent data derived from alpine steppe and alpine meadow, respectively. *** indicates significant correlation between incubation moisture and field moisture at the 0.001 level.

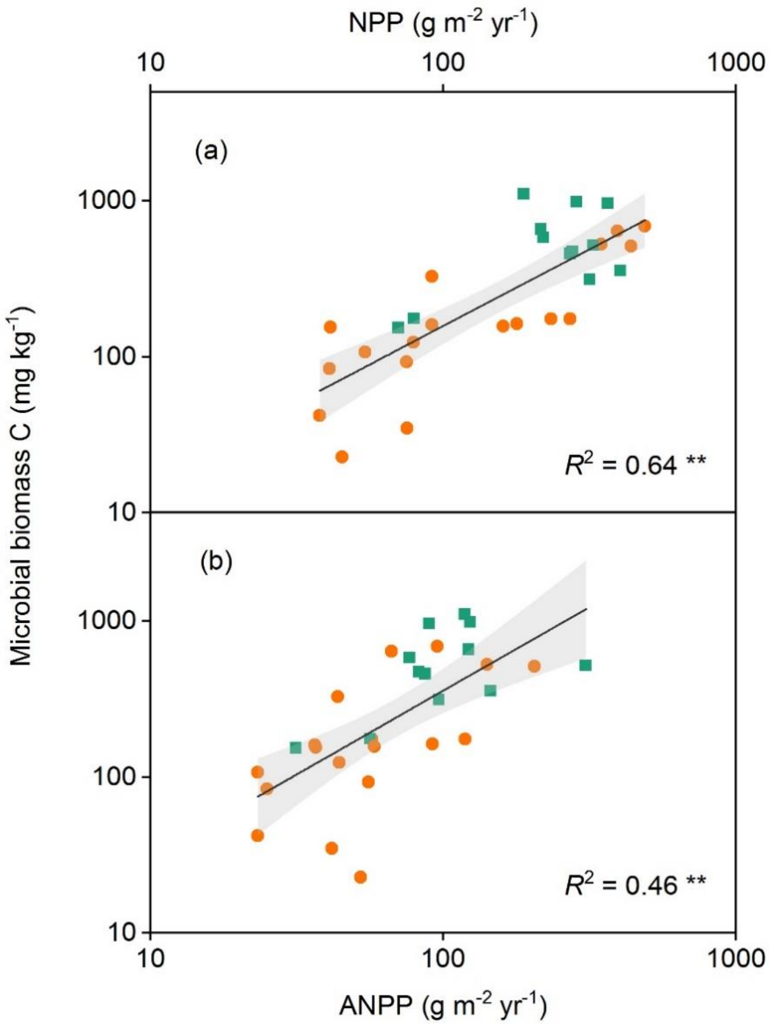

Supplementary Figure 12. Relationship between microbial biomass carbon and net primary productivity (NPP) and aboveground net primary productivity (ANPP). The NPP data (a) were derived from Moderate Resolution Imaging Spectroradiometer (MODIS) (http://modis.gsfc.nasa.gov/), and the ANPP (b) was equal to aboveground biomass obtained from our field survey. A base-10 log scale is used for the x and y axes. The orange dots and green squares represent data derived from alpine steppe and alpine meadow, respectively. ** indicates significant relationships of microbial biomass C with NPP and ANPP at the 0.01 level.

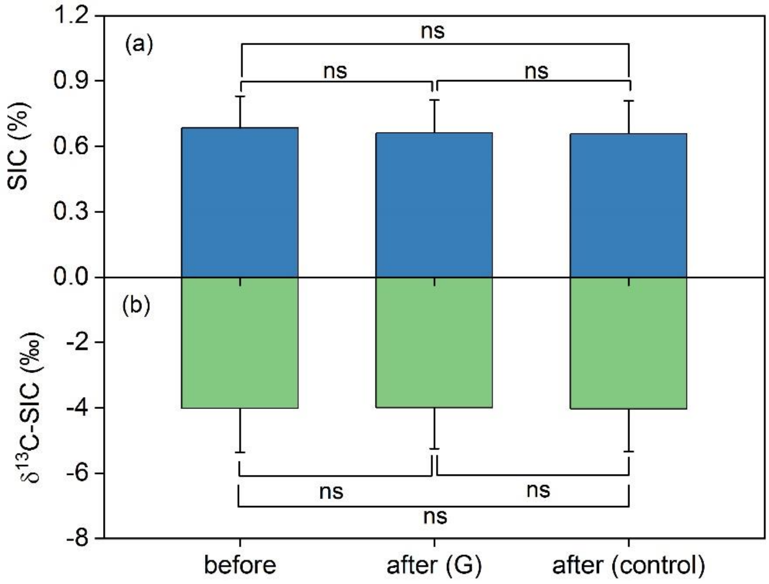

Supplementary Figure 13. Changes in soil inorganic carbon content and its $\delta^{13}C$ value before and after the 65-day incubation experiment. (a) soil inorganic carbon (SIC), (b) $\delta^{13}C$ value of SIC. G, glucose addition treatment. ns, no significant difference at the 0.05 level.

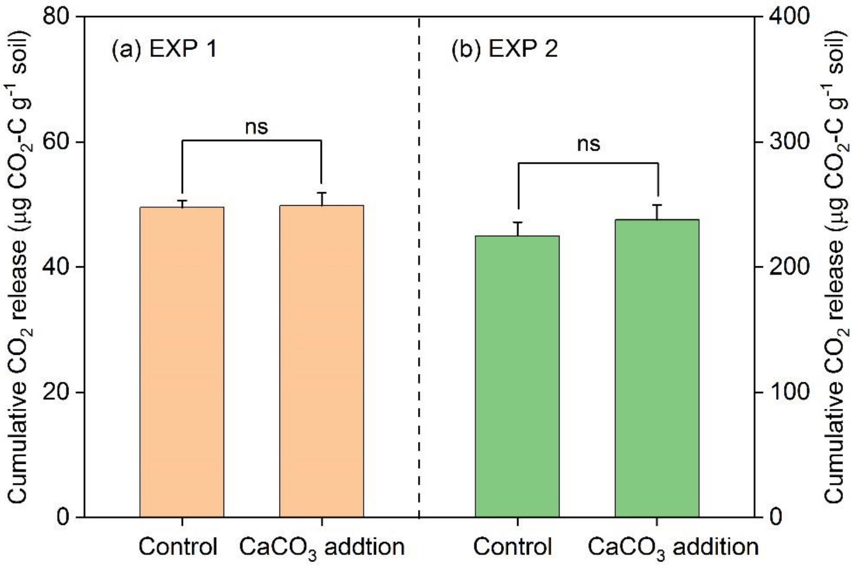

Supplementary Figure 14. Effects of $CaCO_3$ addition on cumulative CO_2 release. The controls in new experiment 1 (a, EXP 1) and experiment 2 (b, EXP 2) are the quartz sand and soil sample without $CaCO_3$ addition, respectively. ns, no significant difference at the 0.05 level.

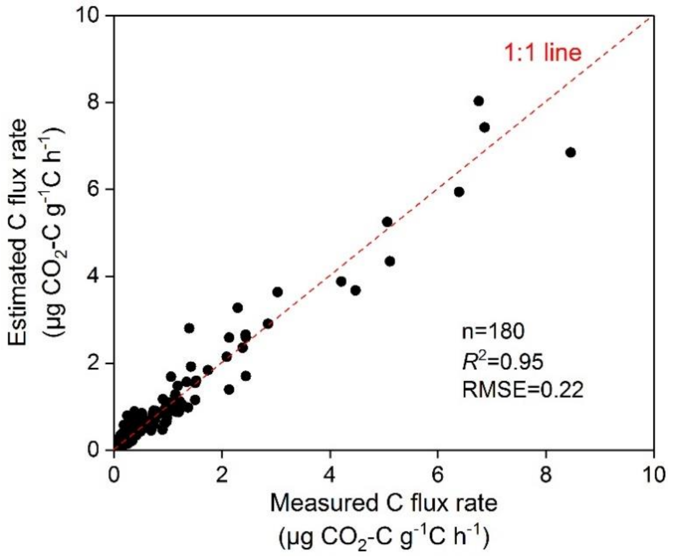

Supplementary Figure 15. Comparison between predicted and measured carbon flux rate from the two-pool carbon decomposition model.

Supplementary Tables

Supplementary Table 1. The background information and the priming effect for the 30 sampling sites along a 2200 km grassland transect on the Tibetan Plateau.

Site No.	Altitude (m)	MAT (ºC)	MAP (mm)	Vegetation type	Dominant species	Soil order	Priming intensity (μg g^{-1} soil dry weight)	Relative priming effect (%)
1	3112	5.08	322	AS	*Stipa sareptana*	Chernozems	96.5 ± 6.3	28.5 ± 1.9
2	3280	2.02	369	AS	*S. sareptana*	Chernozems	66.3 ± 9.9	20.3 ± 3.0
3	3650	-0.43	496	AM	*Kobresia pygmaea*	Cambisols	34.0 ± 10.6	8.0 ± 2.5
4	3830	-0.84	531	AM	*K. pygmaea*	Cambisols	110.5 ± 12.9	37.3 ± 4.1
5	4032	-1.34	534	AM	*Elymus nutans*	Cambisols	112.2 ± 9.8	37.3 ± 3.2
6	4417	-2.93	419	AM	*K. tibetica*	Cambisols	37.1 ±8.4	6.5 ± 1.4
7	4228	-3.25	321	AS	*S. purpurea*	Calcisols	57.1 ± 6.8	15.6 ± 1.9
8	4430	-4.09	508	AS	*S. purpurea*	Cambisols	52.2 ± 5.4	17.9 ± 1.6
9	4162	-0.39	495	AM	*K. pygmaea*	Cambisols	55.5 ± 10.6	13.1 ± 2.2
10	4289	0.15	481	AM	*K. pygmaea*	Cambisols	89.6 ± 9.2	18.2 ± 1.9
11	4161	-1.76	415	AM	*K. pygmaea*	Cambisols	51.7 ± 5.1	10.1 ± 1.0
12	4165	-0.55	338	AS	*S. purpurea*	Cambisols	91.6 ± 4.6	26.5 ± 1.3
13	3224	2.26	324	AS	*S. purpurea*	Cambisols	76.2 ± 7.2	16.3 ± 1.6
14	4637	-4.15	295	AS	*Carex moorcroftii*	Calcisols	15.9 ± 4.5	5.3 ±1.5
15	3137	1.57	314	AS	*S. breviflora*	Kastanozems	113.8 ± 11.7	26.2 ± 2.7
16	3262	0.06	242	AS	*S. sareptana*	Kastanozems	132.5 ± 17.2	30.5 ± 4.0
17	4544	-2.69	89	AS	*C. moorcroftii*	Calcisols	69.5 ± 17.1	13.7 ± 3.4
18	4622	-3.26	307	AM	*K. pygmaea*	Calcisols	56.7 ± 4.5	11.9 ± 1.0

19	4687	-2.84	307	AM	*Festuca ovina*	Calcisols	78.0 ± 5.0	14.9 ± 1.0
20	4639	-1.80	503	AS	*S. purpurea*	Calcisols	63.9 ± 10.2	17.6 ± 2.7
21	4298	-0.35	515	AM	*K. pygmaea*	Cambisols	42.9 ± 5.8	7.9 ± 1.1
22	4748	-0.65	332	AS	*C. moorcroftii*	Calcisols	84.3 ± 4.8	19.1 ± 1.1
23	4590	-0.96	337	AS	*S. purpurea*	Calcisols	28.3 ± 4.4	5.4 ± 0.9
24	4544	0.32	273	AS	*S. purpurea*	Calcisols	67.3 ± 4.0	15.4 ± 0.9
25	4560	0.43	209	AS	*Deyeuxia arundinacea*	Calcisols	26.4 ± 3.2	8.7 ± 1.0
26	4444	0.5	170	AS	*S. caucasica*	Calcisols	43.9 ± 5.7	9.6 ± 1.2
27	4390	0.91	127	AS	*S. tianschanica*	Calcisols	46.0 ± 13.6	16.2 ± 5.0
28	4538	1.12	99	AS	*S. glareosa*	Calcisols	-3.95 ± 0.6	-1.1 ± 0.1
29	4294	1.89	464	AM	*K. pygmaea*	Cambisols	100.5 ± 6.7	18.6 ± 1.2
30	4764	0.08	457	AM	*K. pygmaea*	Cambisols	77.7 ± 4.7	14.2 ± 0.9

Notes: MAT, mean annual temperature; MAP, mean annual precipitation; AS, alpine steppe; AM, alpine meadow. The soil order was derived from the 1:1,000,000 digital soil map of China[6]. The priming data were represented as mean ± 95% confidence interval, which were estimated by the Monte Carlo method[7].

Supplementary Table 2. Prior parameter ranges for C pool partitioning coefficients (*fi*) and decay rates (*ki*) parameter.

Parameter	Description	Lower limit	Upper limit
f_1	proportion of fast carbon pool	0	0.1
k_1	decay rate of fast carbon pool	0	1
k_2	decay rate of slow carbon pool	0	0.01

The proportion of carbon pool is unit less, and the unit of decay rate is day^{-1}.

Parameter ranges were estimated according to previous studies[8, 9].

Supplementary Table 3. Maximum likelihood estimates (MLEs) of posterior probability density functions of model parameters for the 30 study sites.

Site No.	Parameter		
	k_1 ($\times 10^{-2}$)	k_2 ($\times 10^{-5}$)	f_1 ($\times 10^{-2}$)
1	3.50 [3.48, 3.52]	1.25 [1.21, 1.29]	1.14 [1.13, 1.14]
2	2.46 [2.44, 2.48]	2.05 [1.80, 2.30]	1.06 [1.05, 1.06]
3	10.7 [10.3, 11.1]	7.21 [6.99, 7.44]	0.60 [0.58, 0.61]
4	5.03 [4.95, 5.10]	1.53 [1.48, 1.59]	0.47 [0.47, 0.48]
5	4.58 [4.53, 4.62]	1.24 [1.20, 1.29]	0.49 [0.49, 0.50]
6	36.5 [36.4, 36.6]	7.31 [7.27, 7.35]	0.07 [0.07, 0.08]
7	3.88 [3.85, 3.90]	3.17 [3.03, 3.30]	2.90 [2.88, 2.92]
8	4.38 [4.34, 4.41]	0.60 [0.58, 0.62]	0.47 [0.46, 0.47]
9	3.56 [3.52, 3.59]	0.63 [0.60, 0.67]	0.63 [0.62, 0.63]
10	3.62 [3.60, 3.66]	2.70 [2.63, 2.77]	1.11 [1.10, 1.12]
11	4.55 [4.53, 4.57]	1.37 [1.35, 1.38]	0.73 [0.73, 0.74]
12	2.91 [2.87, 2.94]	3.70 [3.49, 3.90]	2.10 [2.08, 2.13]
13	3.84 [3.81, 3.87]	2.69 [2.61, 2.76]	1.61 [1.60, 1.62]
14	3.79 [3.76, 3.81]	10.9 [10.5, 11.2]	4.84 [4.81, 4.88]
15	3.29 [3.26, 3.31]	1.71 [1.65, 1.77]	1.18 [1.18, 1.19]
16	3.59 [3.55, 3.64]	4.10 [3.96, 4.24]	1.48 [1.47, 1.50]
17	34.4 [32.8, 36.0]	59.0 [57.7, 60.3]	1.42 [1.40, 1.44]
18	79.9 [79.3, 80.6]	83.4 [83.1, 83.8]	0.45 [0.45, 0.46]
19	73.6 [72.7, 74.5]	72.0 [71.6, 72.4]	0.45 [0.44, 0.46]
20	4.06 [4.04, 4.07]	2.78 [2.67, 2.88]	4.49 [4.47, 4.50]
21	2.19 [2.14, 2.23]	22.9 [22.7, 23.2]	1.48 [1.45, 1.51]
22	24.1 [23.1, 25.1]	42.6 [42.3, 42.8]	0.44 [0.43, 0.46]
23	42.2 [40.3, 44.1]	93.8 [93.2, 94.5]	1.86 [1.76, 1.97]
24	47.0 [46.1, 47.9]	92.3 [92.0, 92.7]	0.54 [0.53, 0.55]
25	3.59 [3.57, 3.61]	18.5 [18.0, 19.1]	8.15 [8.11, 8.19]
26	2.36 [2.35, 2.37]	37.9 [37.8, 38.0]	9.72 [9.71, 9.74]
27	3.90 [3.87, 3.93]	6.08 [5.87, 6.30]	3.82 [3.79, 3.85]
28	13.8 [13.1, 14.5]	523.6[520.7, 526.5]	2.57 [2.49, 2.64]
29	42.1 [40.5, 43.8]	32.2 [32.0, 32.4]	0.18 [0.17, 0.19]
30	8.64 [8.43, 8.85]	23.9 [23.7, 24.0]	0.41 [0.40, 0.42]

k_1 and k_2 are the decay rates of fast and slow pools (day^{-1}), respectively. f_1 is the proportion of the fast pool. The interquartile range is presented in square brackets.

Supplementary Table 4. Results of principal components analysis (PCA) of plant, soil and microbial properties, and soil organic matter (SOM) stability across the 30 sampling sites.

**Factors**	**PC1**
**Plant properties**	
Relative coverage of grass (%)	-0.76**
Relative coverage of forb (%)	0.31*
Relative coverage of sedge (%)	0.58**
Enhanced Vegetation Index	0.93***
Net primary productivity (NPP, g m^{-2})	0.95***
Aboveground net primary productivity (ANPP, g m^{-2})	0.90***
Amount of C input (mg kg^{-1})	0.82***
Cumulative (%)	59.7
**Soil properties**	
Soil order	-0.62**
pH	-0.61***
Bulk density (g cm^{-3})	-0.82***
Clay content (%)	0.71***
Silt content (%)	0.86***
Sand content (%)	-0.82***
Soil organic C content (g kg^{-1})	0.95***
Total nitrogen content (g kg^{-1})	0.97***
Cumulative (%)	66.0
**Microbial properties**	
Total PLFA (nmol g^{-1})	0.93***
Bacteria (nmol g^{-1})	0.92***
Fungi (nmol g^{-1})	0.74**
Fungi: bacteria ratio	-0.51**
C-acquire enzyme (nmol g^{-1} h^{-1})	0.87***
N-acquire enzyme (nmol g^{-1} h^{-1})	0.72***
P-acquire enzyme (nmol g^{-1} h^{-1})	0.32*
C:N ratio of enzyme	0.71***
N:P ratio of enzyme	0.23*
C:P ratio of enzyme	0.72***
Cumulative (%)	49.67
**SOM stability**	
Carbohydrate (mg g^{-1}OC)	0.72**
Cutin-derived compounds (mg g^{-1}OC)	-0.81***
Suberin-derived compounds (mg g^{-1}OC)	-0.71***
Labile C pool I (%)	0.85***
Labile C pool II (%)	0.81***
Recalcitrant pool (%)	-0.86***

Macroaggregate-associated C (%)	-0.81***
Microaggregate-associated C (%)	0.60**
Clay+silt associated C (%)	0.73***
Molar Fe_d+Al_d /OC ratio	0.96***
Molar Fe_o+Al_o/OC ratio	0.92***
Ca_{exe}/OC ratio	0.87***
Cumulative (%)	65.57

Supplementary References

1. Bossio DA, Scow KM. Impacts of carbon and flooding on soil microbial communities: phospholipid fatty acid profiles and substrate utilization patterns. *Microb Ecol* **35**, 265-278 (1998).
2. Chen LY*, et al.* Determinants of carbon release from the active layer and permafrost deposits on the Tibetan Plateau. *Nat Commun* **7**, 13046 (2016).
3. German DP, Weintraub MN, Grandy AS, Lauber CL, Rinkes ZL, Allison SD. Optimization of hydrolytic and oxidative enzyme methods for ecosystem studies. *Soil Biol Biochem* **43**, 1387-1397 (2011).
4. Zhao L, Wu W, Xu X, Xu Y. Soil organic matter dynamics under different land use in grasslands in Inner Mongolia (northern China). *Biogeosciences* **11**, 5103-5113 (2014).
5. Editorial Committee for Vegetation Map of China (2001). Vegetation Atlas of China. Beijing: Science Press.
6. Shi XZ*, et al.* Soil Database of 1:1,000,000 Digital Soil Survey and Reference System of the Chinese Genetic Soil Classification System. **45**, 129-136 (2004).
7. Luo Z, Wang E, Smith C. Fresh carbon input differentially impacts soil carbon decomposition across natural and managed systems. *Ecology* **96**, 2806-2813 (2015).
8. Schädel C*, et al.* Circumpolar assessment of permafrost C quality and its vulnerability over time using long-term incubation data. *Glob Change Biol* **20**, 641-652 (2014).
9. Schädel C, Luo YQ, Evans RD, Fei SF, Schaeffer SM. Separating soil CO_2 efflux into C-pool-specific decay rates via inverse analysis of soil incubation data. *Oecologia* **171**, 721-732 (2013).
