# Supplementary material for: Regulation of priming effect by soil organic matter stability over a broad geographic scale
Source: Nat Commun. 2019 Nov 8;10:5112. doi: 10.1038/s41467-019-13119-z (PMC6841703; doi:10.1038/s41467-019-13119-z)
Supplement: Supplementary file 3 — Description of Additional Supplementary Files [file 41467_2019_13119_MOESM3_ESM.docx]

**Description of Supplementary Files**

**File Name:** Supplementary Data 1

**Description:** Plant, soil and microbial properties of the 30 sampling sites.
